# Prehospital predicting factors using a decision tree model for patients with witnessed out-of-hospital cardiac arrest and an initial shockable rhythm

**DOI:** 10.1038/s41598-023-43106-w

**Published:** 2023-09-27

**Authors:** Kazuya Tateishi, Yuichi Saito, Yuichi Yasufuku, Atsushi Nakagomi, Hideki Kitahara, Yoshio Kobayashi, Yoshio Tahara, Naohiro Yonemoto, Takanori Ikeda, Naoki Sato, Hiroyuki Okura

**Affiliations:** 1 Department of Cardiovascular Medicine, Chiba University Graduate School of Medicine, 1-8-1 Inohana, Chuo-ku, Chiba, Chiba 260-8677 Japan; 2 Department of Biostatistics and Data Science, Graduate School of Medicine, Osaka University, Osaka, Japan; 3 Department of Cardiovascular Medicine, National Cerebral and Cardiovascular Center, Osaka, Japan; 4 Department of Public Health, Juntendo University School of Medicine Tokyo, Tokyo, Japan; 5 Department of Cardiovascular Medicine, Faculty of Medicine, Toho University, Tokyo, Japan; 6 Cardiovascular Medicine, Kawaguchi Cardiovascular and Respiratory Hospital, Saitama, Japan; 7 Department of Cardiology, Gifu University Graduate School of Medicine, Gifu, Japan

**Keywords:** Medical research, Risk factors

## Abstract

The effect of prehospital factors on favorable neurological outcomes remains unclear in patients with witnessed out-of-hospital cardiac arrest (OHCA) and a shockable rhythm. We developed a decision tree model for these patients by using prehospital factors. Using a nationwide OHCA registry database between 2005 and 2020, we retrospectively analyzed a cohort of 1,930,273 patients, of whom 86,495 with witnessed OHCA and an initial shockable rhythm were included. The primary endpoint was defined as favorable neurological survival (cerebral performance category score of 1 or 2 at 1 month). A decision tree model was developed from randomly selected 77,845 patients (development cohort) and validated in 8650 patients (validation cohort). In the development cohort, the presence of prehospital return of spontaneous circulation was the best predictor of favorable neurological survival, followed by the absence of adrenaline administration and age. The patients were categorized into 9 groups with probabilities of favorable neurological survival ranging from 5.7 to 70.8% (areas under the receiver operating characteristic curve of 0.851 and 0.844 in the development and validation cohorts, respectively). Our model is potentially helpful in stratifying the probability of favorable neurological survival in patients with witnessed OHCA and an initial shockable rhythm.

## Introduction

The prognosis of patients with out-of-hospital cardiac arrest (OHCA) has improved with the development of prehospital and postcardiac arrest care, but OHCA remains a health concern worldwide^1,2^. Recently, practical predictive scoring systems have been developed for evaluating the return of spontaneous circulation (ROSC), overall survival, and favorable neurological survival, providing stratification of OHCAs and facilitating decision-making^3–8^. Bystander witness and initial shockable rhythm are well-known good predictors of resuscitation and favorable neurological survival^9,10^. In addition, early defibrillation plays a crucial role in achieving ROSC in patients with OHCA and a shockable rhythm^11^, in whom prehospital predictors of favorable outcomes may be different from those in the entire OHCA population. The type of patients who would benefit most from early therapeutic strategies, such as defibrillation, in an OHCA setting remains controversial.

The decision tree model developed using recursive partitioning analysis can uniquely provide the probability of a favorable neurological survival and risk stratification of OHCAs^12^, which is readily available in clinical practice. Therefore, we aimed to identify the prehospital factors that would affect favorable neurological survival in patients with witnessed OHCA and an initial shockable rhythm using the decision tree model.

## Methods

### Study design and population (data source)

**Figure 1:**
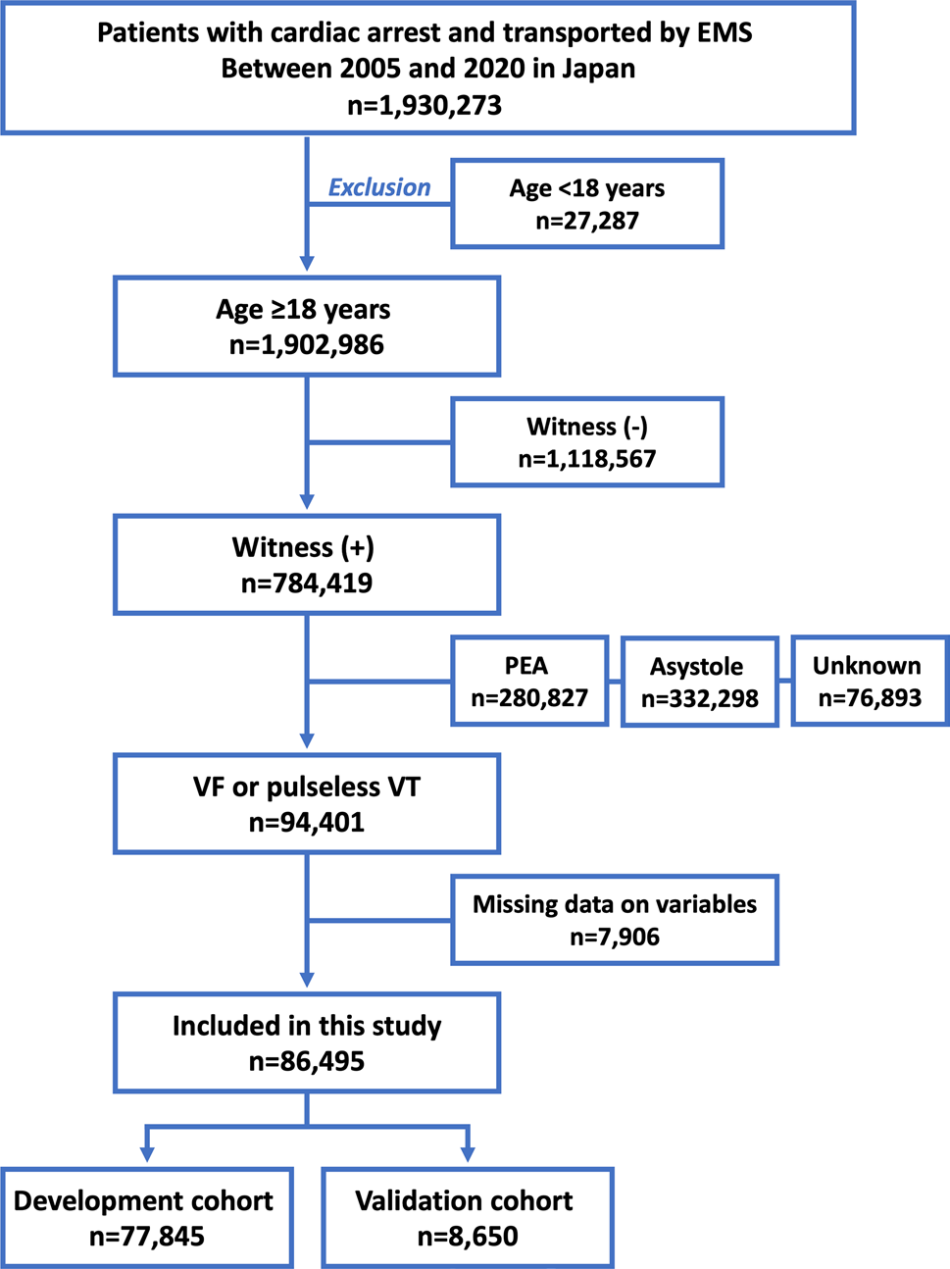
Study flow. EMS, emergency medical services; PEA, pulseless electrical activity; VF, ventricular fibrillation; VT, ventricular tachycardia.

In this retrospective observational study, we used prospectively collected nationwide data from patients with OHCA in Japan based on the Utstein-style template^13,14^. We identified patients aged ≥ 18 years who were transported to a hospital by emergency medical services (EMS) due to OHCA between January 2005 and December 2020. Patients were excluded based on the following criteria: (1) absence of a witness; (2) absence of an initial shockable rhythm; and (3) unknown variable information (Fig. 1). The missing rates of the variables are shown in Table S1.

This study complied with the Declaration of Helsinki regarding human investigations. The Ethics Committee of Chiba University approved this study (unique identifier: #M10316). The requirement for written informed consent has been waived by the Ethics Committee of Chiba University because the data were anonymized.

### Emergency medical service system in Japan

Japan has approximately 800 fire stations with dispatch centers in 47 prefectures. The EMS system is under the supervision of the Fire and Disaster Management Agency (FDMA). EMS personnel, in cooperation with physicians, record data on OHCA patients using a Utstein-style template. The data are then integrated into the National Registry System on the FDMA database server and are checked by the computer system. If any problems are detected, data are sent back to the corresponding fire stations for correction. We utilized anonymous data from the registry, including age, sex, witness, type of initial rhythm, type of bystander, public access automated external defibrillator (AED), number of defibrillation attempts, waveforms of the defibrillator (i.e., monophasic or biphasic), type of airway management device, and adrenaline use. Furthermore, prehospital ROSC, etiology of cardiac arrest, 1-month survival, and neurological function were assessed using the cerebral performance category (CPC) score at 1 month. In addition, information on the time course of collapse, initiation of cardiopulmonary resuscitation (CPR), prehospital ROSC, and arrival at the hospital was obtained.

According to Japanese guidelines^15^, out-of-hospital EMS providers are not allowed to terminate resuscitation in the field. Therefore, all patients with OHCA treated by EMS providers are transported to a hospital. EMS personnel are permitted to perform general medical treatments including the use of AED, basic airway adjuncts, and peripheral intravenous catheters. Furthermore, the insertion of a tracheal tube and the administration of intravenous adrenaline are allowed only under the instructions of a physician in the command center.

### Definition and endpoints

The primary endpoint was a favorable neurological survival at 1 month, which was defined as survival with a CPC score of 1 or 2^16^. We also developed a decision tree model to predict survival at 1 month. Daytime admission was defined as admission to the hospital between 6:00 AM and 5:59 PM. Weekend/holiday admission was defined as admission on a Saturday, Sunday, or Japanese national holiday.

### Statistical analysis

Statistical analysis was performed using the Stata statistical software package version 15.1 (StataCorp LLC, Texas, USA). Continuous variables are expressed as mean ± standard deviation and were compared using Student’s t-test. Categorical data are presented as absolute numbers and percentages and were compared using the chi-square test. Differences were considered statistically significant at *p* < 0.05.

The following 16 prehospital variables were selected for developing a prediction model: age (years old), male (yes or no), collapse witnessed by EMS personnel (yes or no), bystander CPR by citizen (yes or no), chest compression by citizen (yes or no), rescue breathing by citizen (yes or no), AED by citizen (yes or no), biphasic defibrillator (yes or no), the number of defibrillation attempts (times), prehospital use of adrenaline (yes or no), prehospital ROSC (yes or no), collapse-to-CPR time interval (min), collapse-to-first shock time interval (min), collapse-to-hospital arrival time interval (min), daytime admission (yes or no), and weekend/holiday admission (yes or no). Variables with “yes or no” were considered dichotomous.

To develop a decision tree model for the outcomes, we conducted a recursive partitioning analysis using the Gini index^17,18^. Recursive partitioning analysis can provide a branching decision tree by dividing the patient population into subgroups based on the analysis results of the relationship between outcomes and prehospital variables^19^. We initially randomly divided all patients into the validation and development cohorts (a ratio of 1:9). Using the development cohort, tenfold cross-validation was then performed to generate a classification and regression tree. Finally, the predictive ability of the classification and regression tree model was assessed in the development and validation cohorts. To examine the balance of covariate distributions between these cohorts, we calculated the standardized difference.

## Results

Of the 1,930,273 patients registered between 2005 and 2020, 86,495 met the inclusion criteria and were included in the analysis (Fig. 1). We randomly selected a validation cohort (n = 8650) from the entire population and then developed a decision tree model using the rest of the population (n = 77,845). Patient characteristics of the development and validation cohorts are shown in Table 1. No significant differences were found in any of the variables between the two cohorts.

**Table 1:** Patient characteristics.

Variable	All patient (n = 86,495)	Development cohort (n = 77,845)	Validation cohort (n = 8650)	Standardized difference
Age (years)	65.7 ± 15.3	65.7 ± 15.3	65.5 ± 15.5	− 0.01
Male	68,027 (78.7%)	61,515 (78.6%)	6876 (79.5%)	− 0.008
Treatment by citizen	43,412 (50.2%)	39,124 (50.3%)	4288 (49.6%)	0.005
Chest compression by citizen	42,673 (49.3%)	38,460 (49.4%)	4213 (48.7%)	0.005
Rescue breathing by citizen	9976 (11.5%)	8968 (11.5%)	1008 (11.7%)	− 0.002
AED by citizen	3387 (3.9%)	3039 (3.9%)	348 (4.0%)	− 0.002
EMS witness	10,215 (11.8%)	9220 (11.8%)	995 (11.5%)	0.003
Biphasic defibrillation	77,724 (89.9%)	69,965 (89.9%)	7759 (89.7%)	0.002
Defibrillation times	2.5 ± 1.8	2.5 ± 1.8	2.5 ± 1.8	0.000
Adrenaline	19,772 (22.9%)	17,731 (22.8%)	2041 (23.6%)	− 0.007
Prehospital ROSC	28,695 (33.2%)	25,849 (33.2%)	2846 (32.9%)	0.002
Collapse-CPR time	9.4 ± 6.2	9.4 ± 6.2	9.4 ± 6.2	0.000
Collapse-first defibrillation time	11.4 ± 6.8	11.4 ± 6.8	11.4 ± 6.8	0.000
Collapse-hospital arrival time	33.0 ± 14.5	33.0 ± 14.5	33.0 ± 14.7	0.000
Daytime admission	54,248 (62.7%)	48,791 (62.7%)	5457 (63.1%)	− 0.003
Weekend admission	30,286 (35.0%)	27,213 (35.0%)	3073 (35.5%)	− 0.004
Survival at 1-month	27,496 (31.8%)	24,761 (31.8%)	2735 (31.6%)	0.002
Neurologically favorable survival at 1-month	19,190 (22.2%)	17,284 (22.2%)	1906 (22.0%)	0.001

Data are presented as number (%) of patients, mean ± standard deviation.

AED, automated external defibrillator; CPC, cerebral performance category; CPR, cardiopulmonary resuscitation; EMS, emergency medical services; ROSC, return of spontaneous circulation.

**Figure 2:**
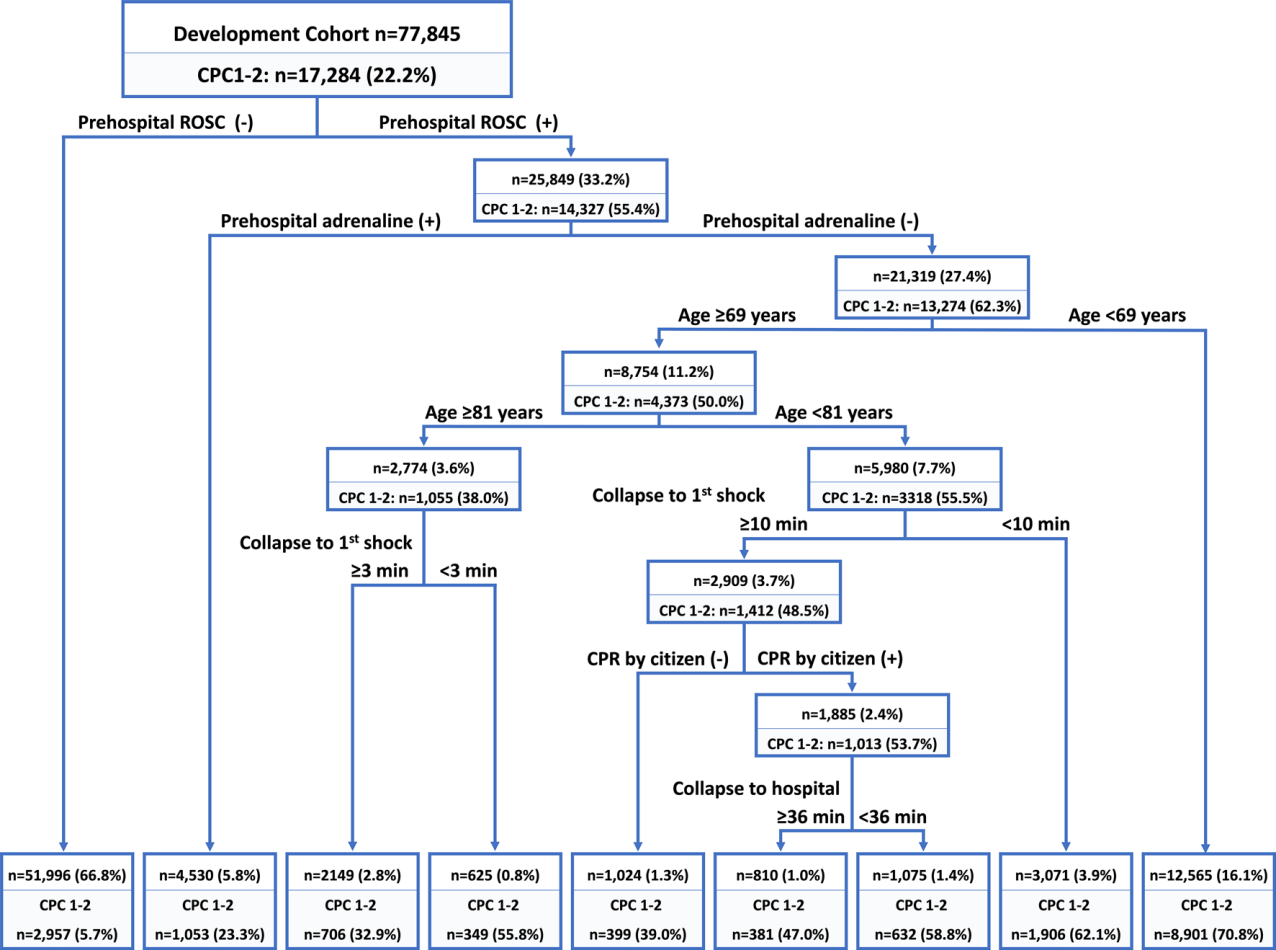
Decision tree model for predicting favorable neurological outcomes at 1 month after cardiac arrest. CPC, cerebral performance category; CPR, cardiopulmonary resuscitation; ROSC, return of spontaneous circulation.

Overall, the proportions of patients with favorable neurological survival were 22.2% and 22.0% in the development and validation cohorts, respectively (Table 1). Figure 2 shows the final decision tree model using recursive partitioning analysis to predict favorable neurological survival at 1 month in the development cohort. This model indicated that the best single classification factor was the presence of prehospital ROSC. The probability of favorable neurological survival for patients without prehospital ROSC was 5.7%. The next best predictor in patients with prehospital ROSC was the absence of prehospital adrenaline use (62.3% predictable favorable neurological survival rate). Patients aged < 69 years provided additional value for patients with prehospital ROSC and without adrenaline use (70.8% predictable favorable neurological survival rate). By contrast, in patients aged ≥ 69 years, a discrimination level of < 81 years old was identified as a good predictor. For patients aged ≥ 81 years, the time from collapse to first shock < 3 min was associated with a better prognosis (55.8% predictable favorable neurological survival rate). Alternatively, for patients aged < 81 years, the time from collapse to first shock < 10 min yielded a better prognosis (62.1% predictable favorable neurological survival rate). Furthermore, if the time from collapse to first shock was delayed by 10 min or more in these patients, the presence of CPR by citizens became the next key factor. Finally, for patients who received CPR by a citizen, earlier transportation (the time from collapse to hospital < 36 min) contributed to a favorable outcome (58.8% predictable favorable neurological survival rate). Our decision tree model can stratify these patients into prediction rates of favorable neurological survival ranging from 5.7 to 70.8% (Fig. 2).

**Figure 3:**
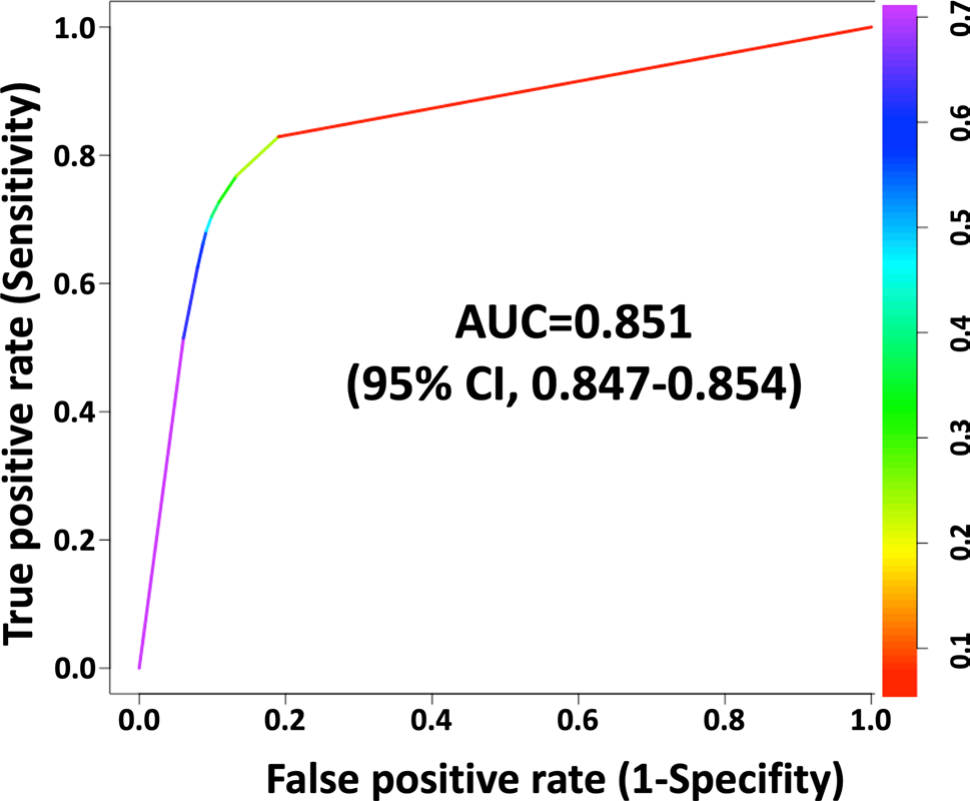
Color-coded ROC curve for this model in the development cohort. The color bar on the right indicates the threshold value of each color. AUC, area under the curve; CI, confidence interval; ROC, receiver operating characteristic.

The area under the receiver operating characteristic curve (AUC) for this model in the development cohort was 0.851 (95% confidence interval [CI], 0.847–0.854) (Fig. 3). This decision tree model was also tested to stratify patients in the validation cohort; the AUC for the validation cohort was 0.844 (95% CI, 0.834–0.855) (Fig. S1). The accuracy, sensitivity, specificity, and AUC of the development and validation cohorts are presented in Table 2. Patient characteristics for favorable neurological survival at 1 month and their counterparts in the development and validation cohorts are shown in Table S2. The classification error rates for the development and validation cohorts are provided in Tables S3 and S4, respectively. The feature importance of this model is shown in Fig. S2.

**Table 2:** The accuracy, sensitivity, specificity, and AUC in the development and validation cohort.

Decision tree models	Accuracy	Sensitivity	Specificity	AUC
Development cohort	0.858	0.682	0.908	0.851
Validation cohort	0.855	0.668	0.908	0.844

AUC, area under the curve.

## Discussion

The main findings of the present study are as follows: (1) patients with OHCA who were < 69 years old and achieved prehospital ROSC without adrenaline use had a high favorable neurological survival rate at 1 month (70.8%) in this population; (2) the time from collapse to first shock was a crucial factor for patients aged ≥ 69 years who achieved prehospital ROSC without adrenaline use; and (3) in addition to the presence of bystander CPR, earlier transportation to hospital had a great effect on the favorable neurological survival rate in patients aged 69–80 years, particularly when the first shock was delayed (≥ 10 min). This prediction model may provide valuable support for decision-making in patients with witnessed OHCA and an initial shockable rhythm in the emergency department.

### Prehospital predictors of outcomes in patients with OHCA

Previous studies proposed useful predictive scoring models for patients with OHCA^3–5^. The ROSC after cardiac arrest score was developed from the German Resuscitation Registry to predict the probability of ROSC with AUCs of 0.71 and 0.73 in the development and validation cohorts, respectively, in patients with OHCA by using sex, age, the presence of bystander witness, initial rhythm, location of cardiac arrest, etiology of cardiac arrest, and time until arrival of professionals^3^. Similarly, using Utstein templates for patient data collection, the Utstein-based ROSC score was developed to identify the probability of ROSC and survival to hospital admission of OHCAs with an AUC of 0.83 by using age, sex, etiology, location, bystander CPR, rhythm, and time to EMS arrival^4^. Recently, in patients with OHCA in Asia, the prehospital ROSC score was developed with an AUC of 0.81 by including variables such as age, time to EMS arrival, initial rhythm, witnessed arrest, and prehospital drug administration^5^.

The aforementioned three risk-scoring models were developed to estimate the probability of ROSC in patients with OHCA, but post-cardiac arrest brain injury is commonly observed in this patient population even when resuscitation is performed^20^. Approximately 80% of patients who are admitted to an intensive care unit after resuscitation from OHCA are comatose^21^, and most of them experience severe neurological disability or death^22^, Thus, the prediction model for neurological outcomes is important to inform patients’ relatives of the correct prognosis and avoid excessive care in patients with irreversible post-cardiac arrest brain injury^20^. In addition, patients with witnessed arrest and/or an initial shockable rhythm have more favorable outcomes than those without^9,10^. Previous studies indicated that early defibrillation is associated with favorable outcomes in patients with witnessed OHCA and a shockable rhythm^11^. Thus, patients with witnessed OHCA and an initial shockable rhythm may have unique prehospital predictors of favorable outcomes. Several prediction models have been developed to estimate favorable neurological survival. The OHCA score, derived from patients with OHCA admitted to a French intensive care unit, can provide a probability of survival with good neurological function, with an AUC of 0.82, using estimated no-flow and low-flow intervals and blood lactate and creatinine levels^23^. However, this prediction model included only patients who achieved successful resuscitation and had blood examination data (i.e., lactate and creatinine levels). In this context, the Cardiac Arrest Survival Score was developed as a simple clinical tool to predict favorable neurological survival at hospital discharge^24^. This prediction model offers the probability of survival with good neurological function, with an AUC of 0.88, using factors such as age, initial rhythm, bystander CPR, adrenaline use, previous disease, place, amiodarone, witness, prehospital ROSC, time from collapse to CPR, and CPR time^24^. These calculation systems may be clinically useful^25^, and decision tree models are also practical for stratifying patient risks and trajectories without a calculator. Goto et al. demonstrated that patients with OHCA can be stratified (from 0.3 to 23.2% of favorable neurological survival probability at 30 days) using four prehospital variables (initial shockable rhythm, age, witnessed arrest, and witnessed by EMS personnel)^12^. Nevertheless, dedicated prediction models for patients with witnessed OHCA and an initial shockable rhythm have not yet been fully evaluated. Although a machine learning-based prognostic model for patients with OHCA and an initial shockable rhythm has been investigated, its clinical applicability may be challenging^26^. Therefore, we aimed to develop a decision tree model for stratifying favorable neurological survival prediction in patients with witnessed OHCA and an initial shockable rhythm using prehospital factors.

### Validation of the present study compared with that of previous studies

In the present study, the presence of prehospital ROSC was the most important factor for achieving a favorable neurological outcome. This result is reasonable because previous studies have shown that prehospital ROSC is one of the strongest predictors of favorable outcomes^9^. The next-best predictor was the absence of prehospital adrenaline administration. In the current guidelines, the administration of prehospital adrenaline for patients with a shockable rhythm is weakly recommended when initial defibrillation attempts have failed^27^. A recent randomized controlled trial and large-scale meta-analysis showed that prehospital adrenaline administration improves the probability of survival to discharge but has no significant effect on favorable neurological outcomes^28,29^. Furthermore, a prospective, nonrandomized, observational propensity analysis reported that the administration of prehospital adrenaline is a significant negative predictor of favorable neurological survival (CPC 1–2: odds ratio 0.31 [95% CI 0.26–0.36]^30^. Although whether prehospital adrenaline is beneficial for patients with OHCA remains controversial, the presence of prehospital adrenaline use is a robust negative factor in achieving favorable neurological survival in this population.

Younger age and earlier defibrillation attempts are well-known risk factors for favorable outcomes^31–33^. In the present study, early defibrillation attempts (< 10 min) provided better neurological prognosis in patients aged 69–80 years old, and very early defibrillation attempts (< 3 min) were associated with neurologically favorable survival in older patients (> 81 years). Early defibrillation is a well-known predictor of favorable prognosis, while very early defibrillation (< 3 min) by EMS may be achieved only in specific situations, such as in patients who experience a cardiac arrest in the presence of EMS. Therefore, the findings on very early defibrillation should be interpreted with caution. Interestingly, this decision tree model suggested that the benefit of bystander CPR on favorable neurological survival was pronounced in patients aged 69–80 years without early defibrillation attempts (≥ 10 min). Furthermore, the factor “bystander CPR by citizen” was selected using recursive partitioning analysis regardless of inputting the categories of “chest compression by citizen,” “rescue breathing by citizen,” and “bystander CPR by citizen,” suggesting that the presence of “first aid” by citizen might be important for favorable neurological survival^34–37^.

Finally, this decision tree model showed that earlier transportation to a hospital considerably affected favorable neurological outcomes in patients receiving bystander CPR by citizens. The effect of transport time on favorable outcomes remains debatable in patients with OHCA^38–40^. A systematic review and meta-analysis reported that paramedic transport time is not significantly different between OHCAs with favorable and those with unfavorable neurological outcomes at hospital discharge (mean difference: + 17 min, 95% CI from − 10.37 to 44.37 min)^38^. However, residual confounding factors may have probably influenced this result^38–40^. Our study suggests that earlier transportation might be beneficial for favorable neurological survival, particularly in patients with witnessed OHCA, an initial shockable rhythm, prehospital ROSC, and bystander CPR, but without early defibrillation attempts.

### Clinical implication

To the best of our knowledge, this is the first decision tree model that was developed particularly for patients with witnessed OHCA and an initial shockable rhythm to predict a favorable neurological prognosis. By using this prediction model, patient risks and prognosis can be promptly stratified based on prehospital factors without a calculator in the emergency room, as opposed to previous scoring models^3–8^, thereby aiding in decision-making for the further application of advanced medical support, such as veno-arterial extracorporeal membrane oxygenation. However, the decision depends on several factors, such as the preference of patient and family members, prognosis at baseline, and medical resources and the accuracy of the present model is not yet established. Therefore, the clinical decision should not be made uniformly. Further, our decision tree model implies that very early defibrillation (< 3 min) may contribute to better neurological prognosis, especially in older patients (> 81 years). In addition, earlier hospital transportation (> 36 min) might be beneficial for favorable neurological survival, particularly in those with prehospital ROSC and who are provided bystander CPR but without early defibrillation attempts. Further studies are warranted to confirm these findings.

### Study limitations

The present study has several limitations. Because we obtained limited information from the FDMA database, important data, such as body mass index, comorbidities, and post-arrest care at a hospital (e.g., mechanical circulatory support, targeted temperature management, and coronary interventional therapies), were not available. In addition, some variables necessary to calculate previous predictable scoring models, such as blood lactate and creatinine levels, were missing. Because we defined short-term favorable neurological survival (at 1 month) as the primary endpoint, our prediction model may not be applicable for predicting long-term outcomes. However, a systematic review reported that long-term neurological outcome scores after OHCA are consistent with short-term outcomes at 30 days^8^. Therefore, our model may also predict long-term outcomes.

## Conclusions

Our decision tree model suggested that prehospital ROSC, absence of adrenaline use in the field, younger age, bystander CPR, earlier defibrillation attempts, and earlier transportation to hospitals were important predictors of favorable neurological survival at 1 month in patients with witnessed OHCA with a shockable rhythm. This prediction model can provide clinicians with risk stratification information in the emergency department.

### Supplementary Information

Supplementary Figure S1.

Supplementary Figure S2.

Supplementary Information 3.

Supplementary Table S1.

Supplementary Table S2.

Supplementary Table S3.

Supplementary Table S4.

## Data Availability

The data that support the findings of this study are available from the Fire and Disaster Management Agency but restrictions apply to the availability of these data, which were used under license for the current study, and so are not publicly available. Data are however available from the authors upon reasonable request and with permission of the Fire and Disaster Management Agency.

## References

[1] Berdowski J, Berg RA, Tijssen JG, Koster RW (2010). Global incidences of out-of-hospital cardiac arrest and survival rates: Systematic review of 67 prospective studies. Resuscitation.

[2] Nichol G (2008). Regional variation in out-of-hospital cardiac arrest incidence and outcome. JAMA.

[3] Gräsner JT (2011). ROSC after cardiac arrest–the RACA score to predict outcome after out-of-hospital cardiac arrest. Eur. Heart J..

[4] Baldi E (2020). An Utstein-based model score to predict survival to hospital admission: the UB-ROSC score. Int. J. Cardiol..

[5] Liu N (2022). Development and validation of an interpretable prehospital return of spontaneous circulation (P-ROSC) score for patients with out-of-hospital cardiac arrest using machine learning: A retrospective study. eClinicalMedicine.

[6] Gue YX (2021). Out-of-hospital cardiac arrest: A systematic review of current risk scores to predict survival. Am. Heart J..

[7] Wong XY (2022). Development and validation of the SARICA score to predict survival after return of spontaneous circulation in out of hospital cardiac arrest using an interpretable machine learning framework. Resuscitation.

[8] Wu W, Chopra A, Ziegler C, McLeod SL, Lin S (2020). Predictive value of hospital discharge neurological outcome scores for long-term neurological status following out-of-hospital cardiac arrest: A systematic review. Resuscitation.

[9] Sasson C, Rogers MA, Dahl J, Kellermann AL (2010). Predictors of survival from out-of-hospital cardiac arrest: A systematic review and meta-analysis. Circ. Cardiovasc. Qual. Outcomes.

[10] Weisfeldt ML, Becker LB (2002). Resuscitation after cardiac arrest: A 3-phase time-sensitive model. JAMA.

[11] Nakahara S (2015). Association of bystander interventions with neurologically intact survival among patients with bystander-witnessed out-of-hospital cardiac arrest in Japan. JAMA.

[12] Goto Y, Maeda T, Goto Y (2013). Decision-tree model for predicting outcomes after out-of-hospital cardiac arrest in the emergency department. Crit. Care.

[13] Cummins RO (1991). Recommended guidelines for uniform reporting of data from out-of-hospital cardiac arrest: The Utstein Style. A statement for health professionals from a task force of the American Heart Association, the European Resuscitation Council, the Heart and Stroke Foundation of Canada, and the Australian Resuscitation Council. Circulation.

[14] Jacobs I (2004). Cardiac arrest and cardiopulmonary resuscitation outcome reports: Update and simplification of the Utstein templates for resuscitation registries: A statement for healthcare professionals from a task force of the International Liaison Committee on Resuscitation (American Heart Association, European Resuscitation Council, Australian Resuscitation Council, New Zealand Resuscitation Council, Heart and Stroke Foundation of Canada, InterAmerican Heart Foundation, Resuscitation Councils of Southern Africa). Circulation.

[15] Nagao K (2016). Duration of prehospital resuscitation efforts after out-of-hospital cardiac arrest. Circulation.

[16] Perkins GD (2015). Cardiac arrest and cardiopulmonary resuscitation outcome reports: update of the Utstein Resuscitation Registry Templates for Out-of-Hospital Cardiac Arrest: A statement for healthcare professionals from a task force of the International Liaison Committee on Resuscitation (American Heart Association, European Resuscitation Council, Australian and New Zealand Council on Resuscitation, Heart and Stroke Foundation of Canada, InterAmerican Heart Foundation, Resuscitation Council of Southern Africa, Resuscitation Council of Asia); and the American Heart Association Emergency Cardiovascular Care Committee and the Council on Cardiopulmonary, Critical Care, Perioperative and Resuscitation. Circulation.

[17] Mingers J (1989). An empirical comparison of selection measures for decision-tree induction. Mach. Learn..

[18] Farris F (2010). The Gini index and measures of inequality. Am. Math Month.

[19] Hess EP (2012). Development of a clinical prediction rule for 30-day cardiac events in emergency department patients with chest pain and possible acute coronary syndrome. Ann. Emerg. Med..

[20] Sandroni C, Cronberg T, Sekhon M (2021). Brain injury after cardiac arrest: Pathophysiology, treatment, and prognosis. Intensive Care Med..

[21] Thomassen A, Wernberg M (1979). Prevalence and prognostic significance of coma after cardiac arrest outside intensive care and coronary units. Acta Anaesthesiol. Scand..

[22] Dragancea I, Rundgren M, Englund E, Friberg H, Cronberg T (2013). The influence of induced hypothermia and delayed prognostication on the mode of death after cardiac arrest. Resuscitation.

[23] Adrie C (2006). Predicting survival with good neurological recovery at hospital admission after successful resuscitation of out-of-hospital cardiac arrest: The OHCA score. Eur. Heart J..

[24] Seewald S (2020). CaRdiac Arrest Survival Score (CRASS)—a tool to predict good neurological outcome after out-of-hospital cardiac arrest. Resuscitation.

[25] Fonarow GC (2005). Risk stratification for in-hospital mortality in acutely decompensated heart failure: Classification and regression tree analysis. JAMA.

[26] Hirano Y, Kondo Y, Sueyoshi K, Okamoto K, Tanaka H (2021). Early outcome prediction for out-of-hospital cardiac arrest with initial shockable rhythm using machine learning models. Resuscitation.

[27] Panchal AR (2020). Part 3: Adult basic and advanced life support: 2020 American Heart Association guidelines for cardiopulmonary resuscitation and emergency cardiovascular care. Circulation.

[28] Perkins GD (2018). A randomized trial of epinephrine in out-of-hospital cardiac arrest. N. Engl. J. Med..

[29] Vargas M, Buonanno P, Iacovazzo C, Servillo G (2019). Epinephrine for out of hospital cardiac arrest: A systematic review and meta-analysis of randomized controlled trials. Resuscitation.

[30] Hagihara A (2012). Prehospital epinephrine use and survival among patients with out-of-hospital cardiac arrest. JAMA.

[31] Kitamura T (2012). Nationwide improvements in survival from out-of-hospital cardiac arrest in Japan. Circulation.

[32] Larsen MP, Eisenberg MS, Cummins RO, Hallstrom AP (1993). Predicting survival from out-of-hospital cardiac arrest: A graphic model. Ann. Emerg. Med..

[33] Swor RA (1995). Bystander CPR, ventricular fibrillation, and survival in witnessed, unmonitored out-of-hospital cardiac arrest. Ann. Emerg. Med..

[34] Iwami T, Kitamura T, Kiyohara K, Kawamura T (2015). Dissemination of chest compression-only cardiopulmonary resuscitation and survival after out-of-hospital cardiac arrest. Circulation.

[35] Kitamura T (2010). Bystander-initiated rescue breathing for out-of-hospital cardiac arrests of noncardiac origin. Circulation.

[36] Ogawa T (2011). Outcomes of chest compression only CPR versus conventional CPR conducted by lay people in patients with out of hospital cardiopulmonary arrest witnessed by bystanders: Nationwide population based observational study. BMJ.

[37] Takei Y (2016). Recruitments of trained citizen volunteering for conventional cardiopulmonary resuscitation are necessary to improve the outcome after out-of-hospital cardiac arrests in remote time-distance area: A nationwide population-based study. Resuscitation.

[38] Geri G (2017). Does transport time of out-of-hospital cardiac arrest patients matter? A systematic review and meta-analysis. Resuscitation.

[39] Spaite DW (2008). The impact of prehospital transport interval on survival in out-of-hospital cardiac arrest: Implications for regionalization of post-resuscitation care. Resuscitation.

[40] Spaite DW (2009). Effect of transport interval on out-of-hospital cardiac arrest survival in the OPALS study: Implications for triaging patients to specialized cardiac arrest centers. Ann. Emerg. Med..

