# Supplementary figures and images for: Prehospital predicting factors using a decision tree model for patients with witnessed out-of-hospital cardiac arrest and an initial shockable rhythm
Source: Sci Rep. 2023 Sep 27;13:16180. doi: 10.1038/s41598-023-43106-w (PMC10533815; doi:10.1038/s41598-023-43106-w)

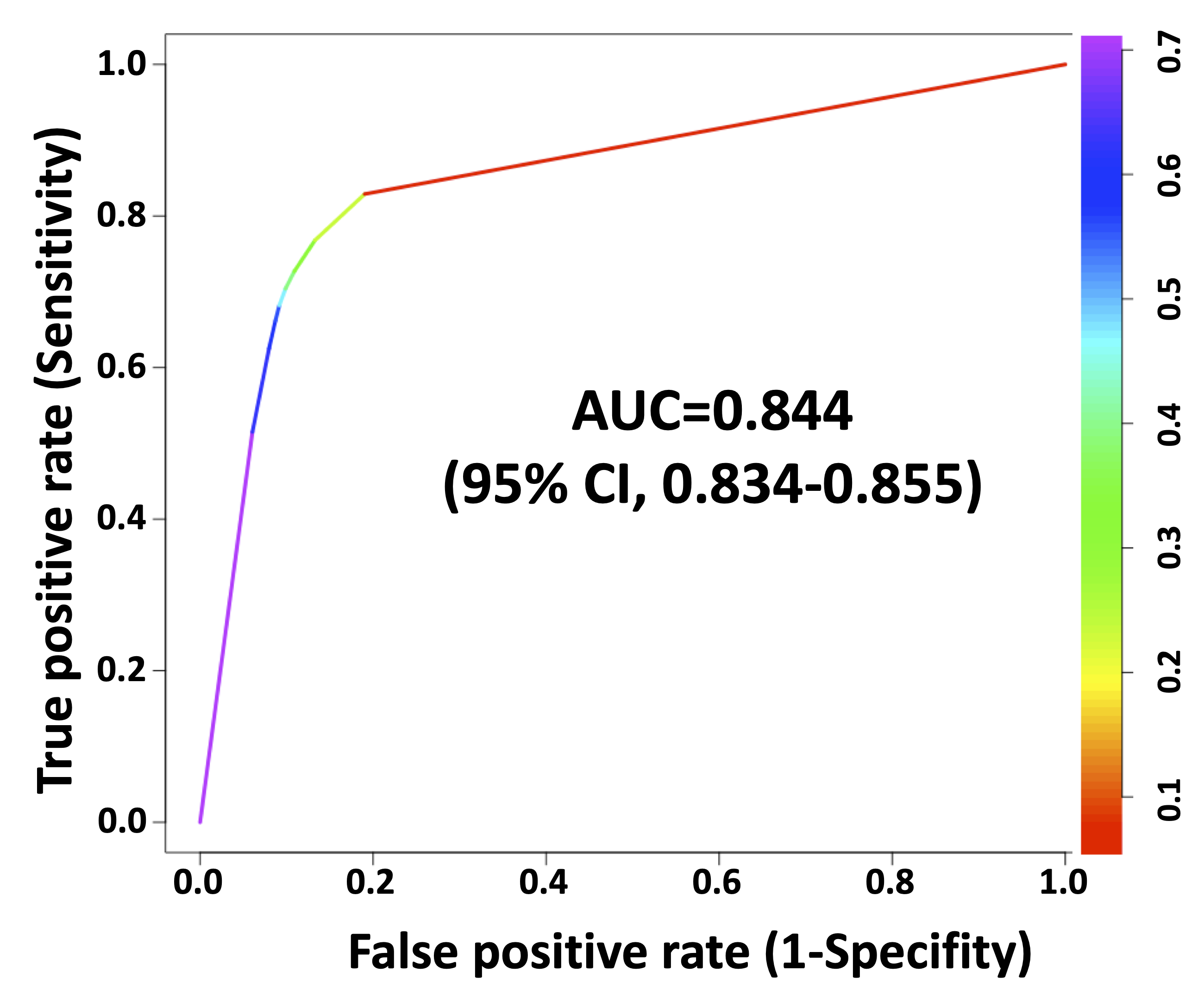

Supplement: Supplementary file 1 — Supplementary Figure S1. [file 41598_2023_43106_MOESM1_ESM.tiff]

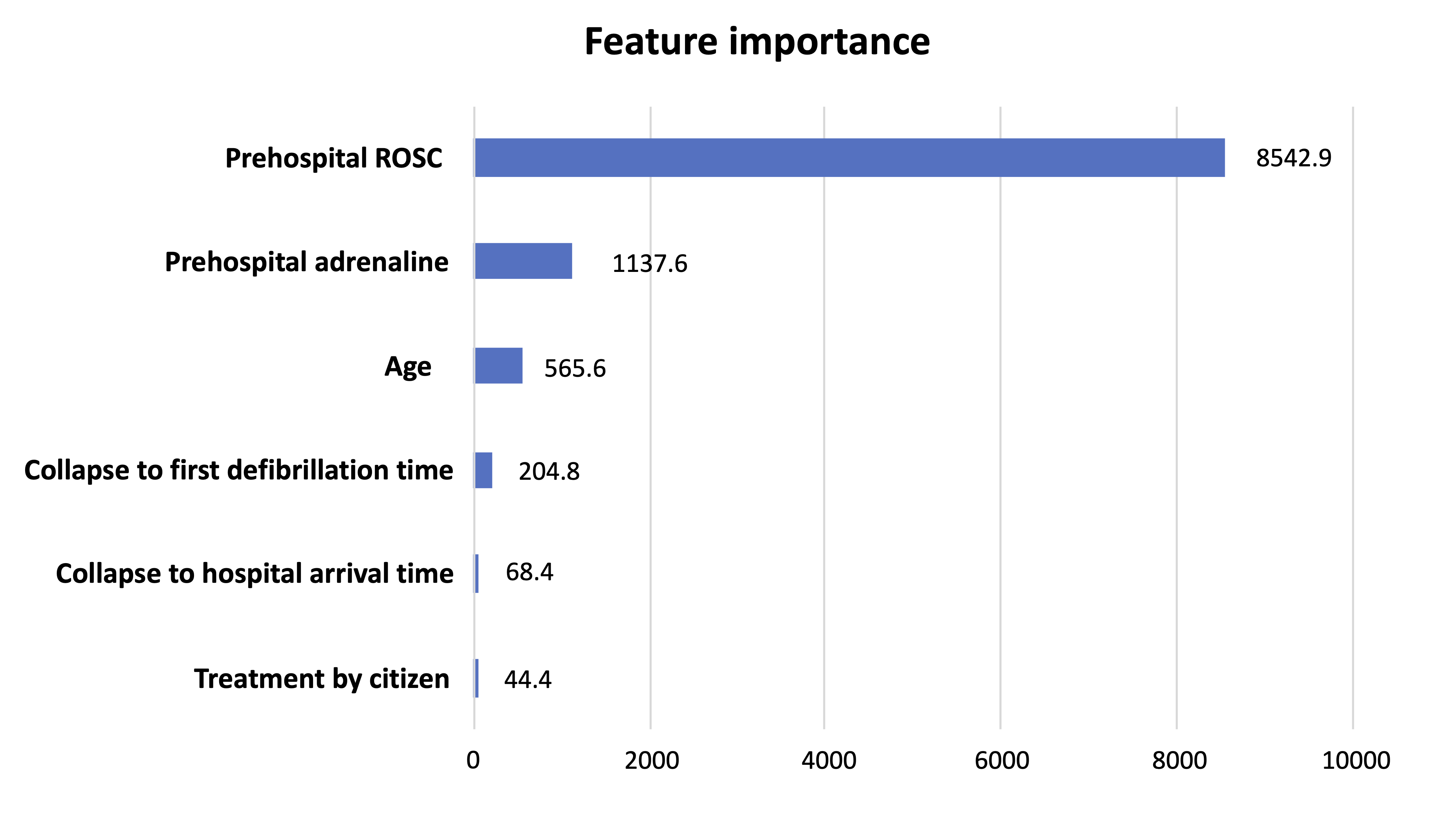

Supplement: Supplementary file 2 — Supplementary Figure S2. [file 41598_2023_43106_MOESM2_ESM.tiff]
