# Supplementary material for: Prehospital predicting factors using a decision tree model for patients with witnessed out-of-hospital cardiac arrest and an initial shockable rhythm
Source: Sci Rep. 2023 Sep 27;13:16180. doi: 10.1038/s41598-023-43106-w (PMC10533815; doi:10.1038/s41598-023-43106-w)
Supplement: Supplementary file 3 — Supplementary Information 3. [file 41598_2023_43106_MOESM3_ESM.docx]

**Figure legends**

**Figure S1.** Color-coded ROC curve for this model in the validation cohort. The color bar on the right indicates the threshold value of each color. Abbreviations: AUC, area under the curve; CI, confidence interval; ROC, receiver operating characteristic.

**Figure S2.** Feature importance of this model. ROSC, return of spontaneous circulation.
