# Supplementary material for: Prehospital predicting factors using a decision tree model for patients with witnessed out-of-hospital cardiac arrest and an initial shockable rhythm
Source: Sci Rep. 2023 Sep 27;13:16180. doi: 10.1038/s41598-023-43106-w (PMC10533815; doi:10.1038/s41598-023-43106-w)
Supplement: Supplementary file 4 — Supplementary Table S1. [file 41598_2023_43106_MOESM4_ESM.docx]

**Table S1. Missing rates of the variables**

| **Variable** | **Missing data** |
| --- | --- |
| Age | 0 (0%) |
| Male | 0 (0%) |
| Treatment by citizen | 162 (0.2%) |
| Chest compression by citizen | 0 (0%) |
| Rescue breathing by citizen | 162 (0.2%) |
| AED by citizen | 0 (0%) |
| EMS witness | 0 (0%) |
| Biphasic defibrillation | 2837 (3.0%) |
| Defibrillation times | 2426 (2.6%) |
| Adrenaline | 0 (0%) |
| Prehospital ROSC | 0 (0%) |
| Collapse-CPR time | 1983 (2.1%) |
| Collapse-first defibrillation time | 6159 (6.5%) |
| Collapse-hospital arrival time | 748 (0.8%) |
| Daytime admission | 0 (0%) |
| Weekend admission | 0 (0%) |

Data are presented as number (%) of patients.

AED, automated external defibrillator; CPR, cardiopulmonary resuscitation; EMS, emergency medical services; ROSC, return of spontaneous circulation.
