# Supplementary material for: Prehospital predicting factors using a decision tree model for patients with witnessed out-of-hospital cardiac arrest and an initial shockable rhythm
Source: Sci Rep. 2023 Sep 27;13:16180. doi: 10.1038/s41598-023-43106-w (PMC10533815; doi:10.1038/s41598-023-43106-w)
Supplement: Supplementary file 5 — Supplementary Table S2. [file 41598_2023_43106_MOESM5_ESM.docx]

**Table S2. Patient characteristics between patients with and without neurologically favorable survival at 1-month**

| **Variable** | **Development cohort** | | **p value** | **Validation cohort** | | **p value** |
| --- | --- | --- | --- | --- | --- | --- |
|  | **CPC1-2 with survival**  **(n=17,284)** | **CPC3-5 or death**  **(n=60,561)** |  | **CPC1-2 with survival**  **(n=1,906)** | **CPC3-5 or death**  **(n=6,744)** |  |
| Age (years) | 60.5±14.8 | 67.2±15.2 | <0.0001 | 60.2±14.8 | 67.0±15.3 | <0.0001 |
| Male | 13868 (80.2%) | 47283 (78.1%) | <0.0001 | 1558 (81.7%) | 5318 (78.9%) | 0.006 |
| Treatment by citizen | 9594 (55.5%) | 29530 (48.8%) | <0.0001 | 1048 (55.0%) | 3240 (48.0%) | <0.0001 |
| Chest compression by citizen | 9431 (54.6%) | 29029 (47.9%) | <0.0001 | 1031 (54.1%) | 3182 (47.2%) | <0.0001 |
| Rescue breathing by citizen | 2184 (12.6%) | 6784 (11.2%) | <0.0001 | 227 (11.9%) | 781 (11.6%) | 0.693 |
| AED by citizen | 1070 (6.2%) | 1969 (3.3%) | <0.0001 | 124 (6.5%) | 224 (3.3%) | <0.0001 |
| EMS witness | 3366 (19.5%) | 5854 (9.7%) | <0.0001 | 377 (19.8%) | 618 (9.2%) | <0.0001 |
| Biphasic defibrillation | 16026 (92.7%) | 60561 (89.1%) | <0.0001 | 1784 (93.6%) | 5975 (88.6%) | <0.0001 |
| Defibrillation times | 2.1±1.5 | 2.6±1.9 | <0.0001 | 2.2±1.6 | 2.6±1.9 | <0.0001 |
| Adrenaline | 1653 (9.6%) | 16078 (26.6%) | <0.0001 | 214 (11.2%) | 1827 (27.1%) | <0.0001 |
| Prehospital ROSC | 14327 (82.9%) | 11522 (19.0%) | <0.0001 | 1565 (82.1%) | 1281 (19.0%) | <0.0001 |
| Collapse-CPR time | 7.4±5.4 | 10.0±6.4 | <0.0001 | 7.5±5.3 | 9.9±6.3 | <0.0001 |
| Collapse-first defibrillation time | 8.9±5.7 | 12.1±7.0 | <0.0001 | 8.9±5.6 | 12.1±7.0 | <0.0001 |
| Collapse-hospital arrival time | 29.8±15.4 | 33.9±14.1 | <0.0001 | 30.0±15.8 | 33.8±14,3 | <0.0001 |
| Daytime admission | 11114 (64.3%) | 37677 (62.2%) | <0.0001 | 1232 (64.4%) | 4225 (62.7) | 0.112 |
| Weekend admission | 6106 (35.3%) | 21107 (34.9%) | 0.248 | 702 (36.8%) | 2371 (35.2%) | 0.178 |

Data are presented as number (%) of patients, mean ± standard deviation.

AED, automated external defibrillator; CPC, cerebral performance category; CPR, cardiopulmonary resuscitation; EMS, emergency medical services; ROSC, return of spontaneous circulation.
