# Supplementary material for: Prehospital predicting factors using a decision tree model for patients with witnessed out-of-hospital cardiac arrest and an initial shockable rhythm
Source: Sci Rep. 2023 Sep 27;13:16180. doi: 10.1038/s41598-023-43106-w (PMC10533815; doi:10.1038/s41598-023-43106-w)
Supplement: Supplementary file 6 — Supplementary Table S3. [file 41598_2023_43106_MOESM6_ESM.docx]

**Table S3. Classification error rate in the development cohort**

| **Actual favorable neurologically survival** | **Predicted favorable neurologically survival** | | | **Total** |
| --- | --- | --- | --- | --- |
|  |  | **Yes** | **No** |  |
|  | **Yes** | 11,788  (15.1%) | 5,496  (7.1) | 17,284 |
|  | **No** | 5,548  (7.1%) | 55,013  (70.7%) | 60,561 |
| **Total** | | 17,336 | 60,509 | 77,845 |
