# Supplementary material for: Prehospital predicting factors using a decision tree model for patients with witnessed out-of-hospital cardiac arrest and an initial shockable rhythm
Source: Sci Rep. 2023 Sep 27;13:16180. doi: 10.1038/s41598-023-43106-w (PMC10533815; doi:10.1038/s41598-023-43106-w)
Supplement: Supplementary file 7 — Supplementary Table S4. [file 41598_2023_43106_MOESM7_ESM.docx]

**Table S4. Classification error rate in the validation cohort**

| **Actual favorable neurologically survival** | **Predicted favorable neurologically survival** | | | **Total** |
| --- | --- | --- | --- | --- |
|  |  | **Yes** | **No** |  |
|  | **Yes** | 1,274  (14.7%) | 632  (7.3) | 1,906 |
|  | **No** | 622  (7.2%) | 6,122  (70.8%) | 6,744 |
| **Total** | | 1,896 | 6,754 | 8,650 |
